# Neuromyelitis Optica Mimicking Multiple Sclerosis: A Case Report and a Comprehensive Review of the Literature

**DOI:** 10.1002/ccr3.71277

**Published:** 2025-10-14

**Authors:** Sunil Thatal, Susmin Karki, Asmita Parajuli, Sweta Bhandari, Bibek K. C., Dibasa Adhikari, Navin Kumar Sah

**Affiliations:** ^1^ B.P. Koirala Institute of Health Sciences Dharan Nepal; ^2^ Maharajgunj Medical Campus Tribhuvan University Institute of Medicine Kathmandu Nepal; ^3^ Department of Internal Medicine B.P. Koirala Institute of Health Sciences Dharan Nepal

**Keywords:** aquaporin‐4 antibody, Devic's disease, multiple myeloma, neuromyelitis optica, NMOSD

## Abstract

Neuromyelitis optica spectrum disorder (NMOSD) may mimic other neurological conditions, including multiple sclerosis (MS). We report a 25‐year‐old woman with hypothyroidism and depression who presented with progressive quadriparesis. Positive aquaporin‐4 antibodies confirmed NMOSD. This case emphasizes the importance of considering NMOSD in patients with atypical demyelinating presentations and highlights the role of early diagnosis and targeted immunotherapy in improving outcomes.

## Graphical abstract

Post‐gadolinium enhancement of the spinal cord seen in NMO.

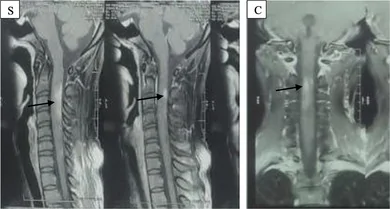

**Summary**

- NMOSD should be considered in patients with MS‐like symptoms; aquaporin‐4 antibody testing is essential to ensure appropriate therapy.

**Abbreviations**
AQP‐4 IgG: aquaporin 4 immunoglobulin G
AZA: azathioprine
C: coronal (C)
CNS: central nervous system
CSF: cerebrospinal Fluid
IPND: international panel for NMO diagnosis
LETM: longitudinally extensive transverse myelitis
MMF: mycophenolate mofetil
MOGAD: myelin oligodendrocyte glycoprotein antibody‐associated disease
MRC: Medical Research Council
MRI: magnetic resonance imaging
MS: multiple Sclerosis
NMOSD: neuromyelitis optica spectrum disorder
S: sagittal
TMS: tumefactive multiple sclerosis
TPE: therapeutic plasma exchange

## 1 Introduction

In 1894, Eugène Devic and his doctorate student Fernand Gault published the first description of neuromyelitis optica derived from neuro‐myélite optique aiguë [1]. For this reason, the illness was originally known as Devic's disease. The prevalence of NMOSD, a rare condition, is thought to vary globally and is 1 per 100,000 among white people, 3.5 per 100,000 among East Asian people, and 10 per 100,000 among black people [2]. Like other autoimmune disorders, there is a strong female predilection for NMOSD [3]. While the cases of multiple sclerosis (MS) worldwide are approximately 2.3 million people, the highest incidence is found in North America (140 cases per 100,000) and Europe (108 cases per 100,000); the lowest incidence is found in East Asia (2.2 cases per 100,000) and sub‐Saharan Africa (2.1 cases per 100,000). In the UK, there are about 120,000 MS patients overall [4].

Neuromyelitis optica spectrum disorder (NMOSD) is primarily characterized by the presence of autoantibodies against aquaporin‐4 (AQP4‐IgG), which are pathogenic and play a central role in its pathophysiology. These antibodies target the AQP4 water channels located on astrocytes, leading to astrocytopathy and subsequent neuroinflammation. The binding of AQP4‐IgG to these channels triggers the classical complement cascade, resulting in astrocyte injury, demyelination, and neuronal damage through both complement‐dependent and independent mechanisms [2, 5, 6]. The pathophysiological process involves the activation of B and T cells, innate immune cells, and the release of pro‐inflammatory cytokines, which contribute to lesion formation in the central nervous system (CNS). This cascade of immune responses leads to the characteristic clinical manifestations of NMOSD, such as optic neuritis, longitudinally extensive transverse myelitis, and area postrema syndrome [2, 5]. Unlike most MS patients, whose first manifestation usually occurs between the ages of 20 and 40 years [2, 6, 7], the median age of onset in NMOSD is 39 years [8]. Wingerchuk et al.'s study proposed the criteria for defining the syndrome in 2006 [8]. The International Panel for NMO Diagnosis (IPND) suggested renaming NMO as neuromyelitis optica spectrum disease (NMOSD). It released an updated set of diagnostic criteria, which comprises core clinical characteristics, magnetic resonance imaging (MRI) neuroimaging features, aquaporin 4 immunoglobulin G (AQP‐4 IgG) status, etc. [7]. The presentation of NMO can be a monophasic or relapsing–remitting course [9]. Cerebrospinal fluid (CSF) findings help distinguish NMOSD from MS. For instance, NMOSD shows pleocytosis, elevated IL‐6 in CSF, and elevated serum AQP4‐IgG in most instances [7, 8, 10, 11, 12, 13, 14]. Treatment of acute attacks of NMOSD includes high‐dose steroids, while therapeutic plasma exchange for refractory cases and long‐term immunosuppression is needed to prevent relapse and severity [15, 16, 17, 18]. In patients with NMO, intensive rehabilitation programs and specific exercises have a small impact on rehabilitation [19, 20]. The case report aims to highlight the importance of distinguishing NMOSD from other conditions, as well as the role of early recognition and targeted immunotherapy, which are essential to prevent long‐term disability and optimize patient outcomes.

## 2 Case History and Examination

A 25‐year‐old female, right‐handed, with hypothyroidism and moderate depression, presented to the emergency department with complaints of weakness in bilateral upper and lower limbs for 10 days. At first, she developed weakness in her right hand, which was associated with difficulty in doing normal daily activities, associated with pain, tingling, and numbness that gradually progressed over three days to the right lower limb, left lower limb, and then to the left upper limb. The patient has been bedridden for 5 days. There is no history of fever, trauma, loss of consciousness, nausea, vomiting, headache, bowel and bladder involvement, or h/o jerky movement. She had a history of atraumatic paraplegia one year back, for which the patient did not seek medical help, and according to the patient, the paraplegia showed gradual improvement.

Her general physical condition and vitals are stable. On neurological examination, her higher mental function is intact. She had no signs of meningeal irritation. Her motor examinations revealed normal tone and bulk in all four limbs. The power in all four limbs was 3/5, according to the Medical Research Council (MRC). Reflexes are 3+ in all four limbs. Clonus is absent. She has decreased sensation in all four limbs. The cranial nerves, including the optic nerve, were normal, and cerebellar examination could not be performed due to quadriparesis, and there was an absence of nystagmus. Other systemic examinations were within normal limits. The quadriparesis involving the distal and proximal muscles, with sensory involvement, without bowel and bladder involvement, and without areflexia, led to the differential diagnosis of Neuromyelitis Optica with myelopathy, multiple myeloma, etc.

## 3 Methods

**FIGURE 1:**
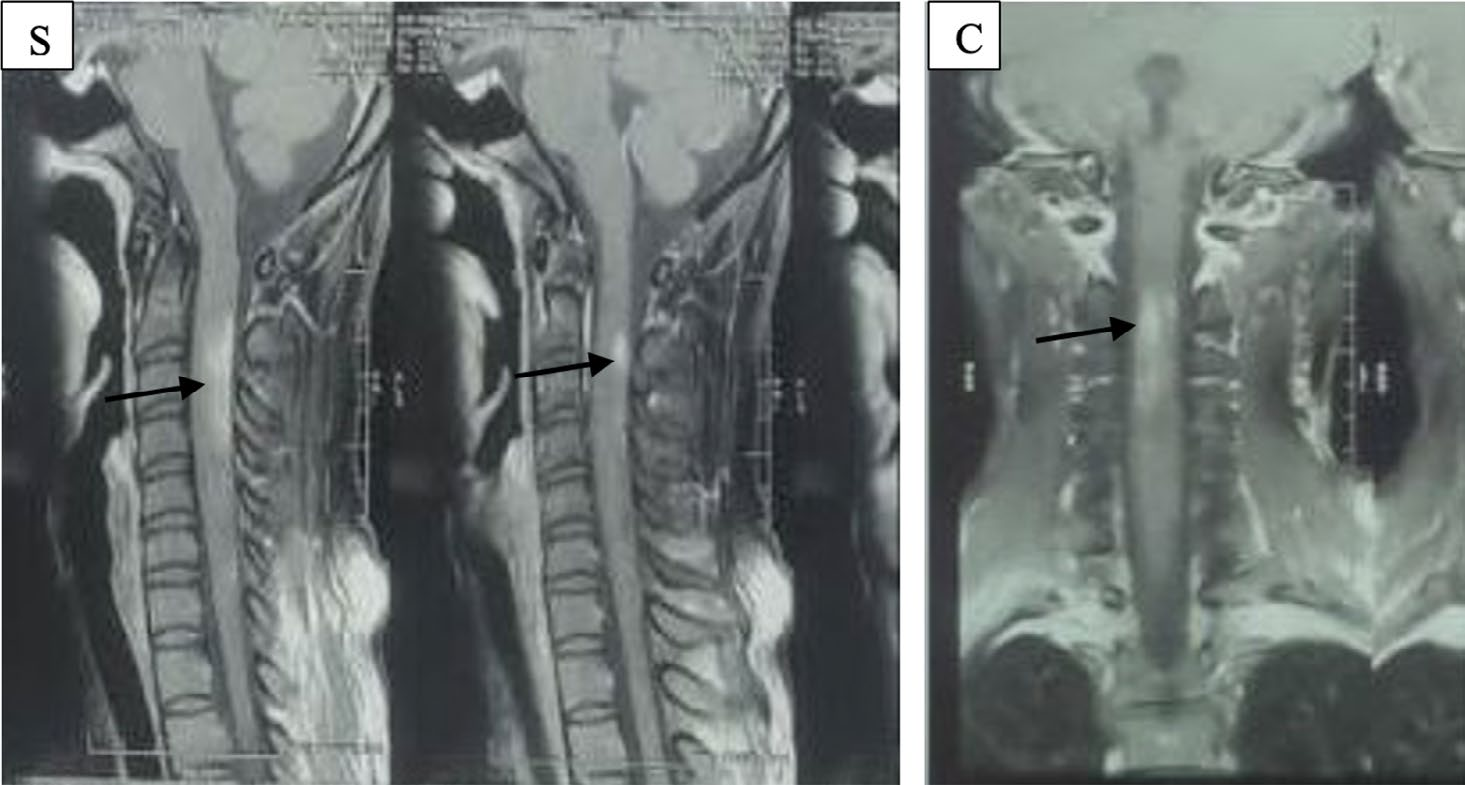
The sagittal (S) and coronal (C) T1 post‐gadolinium image shows a short segment area of enhancement extending from the C3–C6 vertebral level, predominantly involving the posterior segment of the cord (arrow).

**FIGURE 2:**
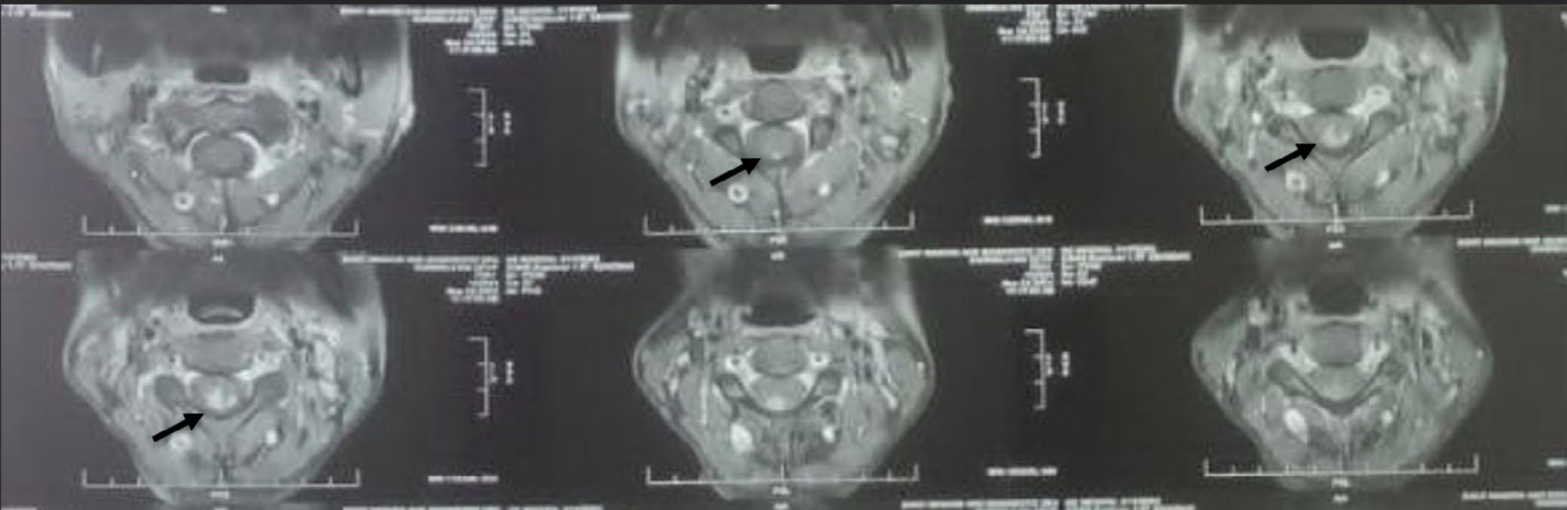
The axial image through the C3 and C4 level shows an area of enhancement in the post‐contrast image predominantly involving the posterior segments (arrow). No evidence of cord swelling is seen.

Furthermore, the T1 post‐gadolinium coronal (C) and sagittal (S) images showed a short segment area of enhancement extending from the C3–C6 vertebral level, predominantly involving the posterior segment of the cord (Figure 1). The axial image through C3 and C4 levels shows the area of enhancement in the post‐contrast image predominantly involving the posterior segments with no evidence of cord swelling (Figure 2). Then, from MRI, we made a differential diagnosis of Multiple Sclerosis, Neuromyelitis Optica Spectrum Disorder, Myelin oligodendrocyte glycoprotein antibody‐associated disease (MOGAD), Transverse myelitis, etc. To rule out the differentials, we performed an anti‐NMO antibody/aquaporin 4 test, which was positive with a titer of 1:10. Our final diagnosis was Neuromyelitis Optica Spectrum Disorder (aquaporin 4 positive). She was treated with Rituximab and methylprednisolone. The patient's neurological features, including motor, sensory, cranial, and higher mental function, improved with time, and she is currently receiving a scheduled cycle of Rituximab.

## 4 Conclusion and Results

The presented case of a young female with Neuromyelitis Optica Spectrum Disorder (aquaporin‐4 positive) demonstrates an atypical progression of quadriparesis involving sensory deficits without bowel and bladder involvement. Imaging studies and serological confirmation were critical in establishing the diagnosis. This case underscores the importance of considering NMOSD in patients presenting with atypical quadriparesis, as timely diagnosis and targeted immunotherapy can significantly improve outcomes.

## 5 Discussion

Neuromyelitis optica (NMO), or Devic's disease, was redefined by the International Panel for NMO Diagnosis (IPND) as neuromyelitis optica spectrum disorder (NMOSD), with revised consensus criteria published in 2015, based on characteristic clinical features, AQP4‐IgG seropositivity, and MRI findings [7].

The inflammation of the spinal cord (myelitis) and one or both optic nerves (optic neuritis) is the hallmark of neuromyelitis optica. Hence, traditionally, NMO was regarded as an opticospinal disease. It typically follows a relapsing and remitting course, but no progression between the attacks. However, the monophasic form can also occur [21]. Neuromyelitis optica spectrum disorder (NMOSD) can be challenging to diagnose due to its overlapping clinical features with other conditions. Several disorders can mimic NMOSD, leading to potential misdiagnosis. Key conditions that may resemble NMOSD include: Myelin Oligodendrocyte Glycoprotein Antibody‐Associated Disease (MOGAD) is another demyelinating disorder that can present with optic neuritis and myelitis, similar to NMOSD. It is distinguished by the presence of MOG antibodies and has distinct neuroimaging findings, such as acute disseminated encephalomyelitis [10]. A variety of non‐demyelinating conditions can mimic NMOSD, including genetic/metabolic disorders, neoplasms, infections, vascular disorders, spondylosis, and other immune‐mediated disorders. These conditions may present with similar clinical syndromes, such as myelopathy and optic neuropathy, but often lack specific features of NMOSD, such as cerebrospinal fluid pleocytosis and response to immunotherapy [22]. Although rare, NMOSD can present with symptoms such as hemiparesis, which may initially be mistaken for a stroke. This highlights the importance of considering NMOSD in differential diagnoses, especially when typical stroke treatments do not lead to improvement [23]. NMOSD can sometimes mimic infectious meningomyelitis, particularly when cerebrospinal fluid analysis shows elevated cell counts and protein levels. This can complicate the diagnostic process, necessitating careful evaluation and testing for aquaporin‐4 antibodies [24].

Multiple Sclerosis (MS) is a well‐known demyelinating disorder that shares clinical features with NMOSD, such as optic neuritis and myelitis. However, MS typically presents with shorter spinal cord lesions and different MRI characteristics compared to NMOSD [22, 25]. The acute episodes of transverse myelitis frequently result in limb weakness, sensory loss, and bladder dysfunction, or bilateral or quickly sequential optic neuritis, which causes significant painful visual loss and usually has a recurrent history [7]. In some patients, optic neuritis and transverse myelitis occur concurrently; in others, clinical episodes are separated by a variable time delay. Most attacks occur over several days, and recovery times range from weeks to months. Though not all clinical symptoms are unique to a given disease, some are very distinctive. In addition to optic neuritis and transverse myelitis, other manifestations of NMO include area postrema syndrome: nausea and vomiting or hiccups, sometimes intractable, occur with an incidence of 16% to 43% in NMOSD. The brainstem involvement may lead to acute neurogenic respiratory failure and death; symptoms related to hypothalamic lesions may include symptomatic narcolepsy or excessive daytime sleepiness, obesity, and various autonomic manifestations such as hypotension, bradycardia, and hypothermia [26]. Hyponatremia secondary to the syndrome of inappropriate antidiuretic hormone secretion or to cerebral salt‐wasting syndrome has been associated with seropositive AQP4 antibody NMOSD [27, 28]. Patients who are aquaporin‐4 (AQP4) antibody‐positive and experience intermittent or repeated episodes of optic neuritis, myelitis, or brain or brainstem syndromes, many of which are difficult to differentiate from multiple sclerosis (MS), have been added to the NMOSD spectrum over time. A minority of children with NMOSD have brain involvement at presentation associated with clinical features of encephalopathy, seizures, and/or lesions on brain MRI resembling those typically seen with MS or acute disseminated encephalomyelitis [29]. NMOSD has a relapsing course in 90% or more of cases [30]. Relapse occurs within the first year following an initial event in 60% of patients and within three years in 90% [31]. As a rule, severe residual deficits follow initial and subsequent attacks, leading to rapid development of disability due to blindness and paraplegia within five years [32, 33]. Unlike MS, a secondary progressive phase of the disease is rare, and disability is associated with specific attacks. Patients with cerebral presentations may have continued brain attacks without the involvement of the optic nerves or spinal cord [34]. Compared to MS, NMOSD is significantly more commonly linked to both organ‐specific and non‐organ‐specific autoimmunity. Other than CNS, NMOSD affects diverse organs such as the kidney, placenta, muscles, etc., and is associated with neoplastic and autoimmune conditions at a higher frequency compared to MS [35, 36, 37, 38, 39, 40].

NMOSD is evaluated by clinical examination, serologic testing for the AQP4‐IgG, and MRI with (and without) gadolinium. Characteristics of MRI findings of NMOSD include involvement of the optic nerve in the form of long‐length/posterior‐chiasmal lesions and longitudinally extensive transverse myelitis (LETM) (greater than or equivalent to three vertebral segments of the spinal cord, mainly involving central/gray matter, presenting as a T1 hypointensity). In the brain, lesions affecting the corticospinal pathways and peri‐ependymal lesions surrounding the ventricular system (wide‐based along the ependymal lining) are present [34]. On the other hand, extensive demyelinating lesions on MRI are also possible in MS patients; nevertheless, these lesions are characterized by partial or nearly full (ovoid) ring enhancement [41, 42]. This type of MS is known as “tumefactive multiple sclerosis (TMS),” in which a single intracranial lesion with a diameter greater than 2.0 cm is a typical MRI finding. Multiple lesions have also been observed, which present a diagnostic conundrum for radiologists and physicians alike. Features suggestive of MS in MRI include short, often multiple, peripheral, asymmetrical, posterior predominant lesions of the spinal cord along with classical “Dawson fingers” (perpendicular to ventricles), U‐fiber involvement, lesions of the inferior lateral ventricle, and temporal lobe of the brain [34]. These radiological characteristics are therapeutically valuable since treatment approaches vary for MS and NMOSD patients, and a false positive increases the risk of significantly higher morbidity in these individuals [7]. In our case, MRI features suggest MS. The cerebrospinal fluid (CSF) analysis was positive for oligoclonal IgG bands, which supports MS, but we did not perform a CSF analysis. The transmembrane protein aquaporin‐4 (AQP4), which promotes water transport in the central nervous system, is the target of the NMO antibody. The brainstem, periventricular white matter, optic nerves, hypothalamus, and spinal cord gray matter all have high levels of AQP4 expression. Following the identification of serum antibodies (Ab) against aquaporin‐4 (AQP4), neuromyelitis optica (NMO), an inflammatory CNS illness, is now recognized as a separate entity from multiple sclerosis (MS) [43]. Only 70% of NMO patients, however, had positive AQP4 antibody levels. The antibody is not expressed by the remaining 30% [44]. While several AQP4‐IgG‐negative NMOSD patients have been found to be MOG‐IgG+ in more recent times due to the ability to detect serum myelin oligodendrocyte glycoprotein IgG (MOG‐IgG). Even while AQP4‐ or MOG‐IgG is typically associated with NMOSD, a small percentage of individuals are doubly seronegative and lack a diagnostic marker. Other CNS inflammatory illnesses, such as sarcoidosis and multiple sclerosis (MS), can occasionally develop in these patients [45, 46]. For medical professionals, this incredibly precise biomarker provided a means of validating the diagnosis of NMO and distinguishing it from multiple sclerosis, a far more prevalent relapsing–remitting autoimmune disease affecting the central nervous system. However, the co‐occurrence of NMOSD and MS in siblings of the Sardinian family has also been reported [47]. NMOSD is less linked to the presence of oligoclonal bands and more likely to induce pleocytosis (incidence roughly 35% in NMOSD), especially in the presence of neutrophils or eosinophils [7]. Additionally, NMOSD patients exhibit elevated CSF IL‐6, a finding not seen in MS patients [10].

There is no cure for NMO. Since MS is a demyelinating disorder and NMO is largely an astrocytopathy, the treatments for these two disorders need to be differentiated to start the right treatment as soon as possible and reduce the significant morbidity associated with these disorders. Therefore, it is crucial to understand the location of lesions and their MRI characterization before starting treatment [43]. A dozen efficacious immunosuppressive and immunomodulatory therapies have been implemented to avert NMOSD relapses. Clinical attacks of NMOSD should be aggressively treated, and an early and timely escalation of therapy is highly suggested, as the long‐term prognosis of NMOSD is most importantly dependent on successful treatment of the attacks [48].

The most promising strategies are interleukin‐6 receptor blocking (tocilizumab), complement cascade inhibition (eculizumab), and B‐cell depletion (rituximab). Additionally, efforts are being made to target autoimmunity in NMO more precisely by developing tolerance to AQP4 [49] or infusing “aquaporumab,” which would selectively block AQP4 antibodies. It is hoped that these developments will eventually result in a cure for this debilitating illness in addition to helping a growing number of NMOSD patients achieve long‐term remission [35]. Studies have demonstrated that intravenous methylprednisolone pulse therapy significantly reduces clinical impairment and enhances the preservation of the thickness of the retinal nerve fiber layer [50, 51]. For both seropositive and seronegative patients, five to seven cycles of therapeutic plasma exchange (TPE) alleviate attack‐related impairment when remission is absent or insufficient, albeit other kinds of auto‐Ab may still be present [52, 53]. In our case, methylprednisolone and Rituximab were used, and other new treatment modalities are unavailable in our country. Intravenous pulse methylprednisolone therapy is the treatment for acute attacks in a seropositive NMO. This is followed by oral prednisolone therapy, which is reduced over several months after symptom resolution to prevent complications and recurrence. In patients who did not respond well to corticosteroid medication, intravenous immunoglobulin or plasmapheresis are used as treatments. Due to the significant relapse rate associated with NMO, ongoing immunosuppressive medication with azathioprine and/or low‐dose prednisolone is necessary. Seronegative NMO patients receive treatment in a manner akin to that of seropositive patients. A poor prognosis was linked to patients who experienced repeated relapses during the first two years of follow‐up, were extremely ill after the initial attack, presented late, and had concurrent systemic lupus erythematosus or other autoimmune disorders [54]. Treatment of an acute attack of NMOSD includes high‐dose steroids, therapeutic plasma exchange for refractory cases, and long‐term immunosuppression [16, 17, 18]. To delay the time to relapse, reduce the severity of future attacks, and minimize permanent disability, long‐term immunosuppression (e.g., Rituximab, MMF, AZA) should be started early [15]. The use of MS medications should be cautious in seronegative NMO [55] since they may worsen antibody‐mediated disorders like AQP4‐IgG+ NMOSD or be ineffective in MOGAD (MOG antibody‐associated disease) [56]. Our case had AQP4‐IgG positive in her serum. Serum AQP4‐IgG testing is a vital part of the diagnostic evaluation. In our case, we diagnosed the patient based on AQP4‐IgG positive NMOSD after the first attack.

The course and prognosis of NMOSD can vary. Relapsing (80% to 90%) or monophasic is the nature of this illness. Certain patients experience recurrent episodes leading to chronic impairment. According to study data, 7% of patients do not show any recovery at all, while up to 22% of patients recuperate completely. A worse prognosis applies to those with relapsing NMOSD. Three percent of patients with monophasic disease incidence and around 5% of relapsing illness patients have either monoplegia or paraplegia. While only 22% of patients with monophasic disease are blind in both eyes, about 60% of patients with relapsing disease are blind in one or both eyes [19, 20]. NMO differs from traditional relapsing–remitting multiple sclerosis (MS) in terms of imaging characteristics, therapeutic response, biomarkers, and pathophysiology.

## 6 Limitations

This case report has several limitations. As a single patient observation, its findings cannot be generalized to the broader population with Neuromyelitis Optica Spectrum Disorder (NMOSD). Importantly, both MRI imaging and anti‐aquaporin‐4 antibody testing were not available at our center and had to be conducted at external facilities. This highlights a delay in diagnosis and the challenges faced in resource‐limited settings. Additionally, cerebrospinal fluid analysis was not performed, and long‐term follow‐up data are currently lacking. The patient also had a similar episode a year prior that went undocumented, limiting our understanding of potential disease recurrence.

## Author Contributions

**Sunil Thatal:** conceptualization, data curation, formal analysis, investigation, methodology, project administration, resources, software, supervision, validation, visualization, writing – original draft, writing – review and editing. **Susmin Karki:** conceptualization, data curation, formal analysis, investigation, methodology, project administration, resources, software, supervision, validation, visualization, writing – original draft, writing – review and editing. **Asmita Parajuli:** conceptualization, data curation, formal analysis, investigation, methodology, project administration, resources, software, supervision, validation, visualization, writing – original draft, writing – review and editing. **Sweta Bhandari:** conceptualization, data curation, formal analysis, investigation, methodology, project administration, resources, software, supervision, validation, visualization, writing – original draft, writing – review and editing. **Bibek K. C.:** supervision, writing – original draft, writing – review and editing. **Dibasa Adhikari:** supervision, writing – original draft, writing – review and editing. **Navin Kumar Sah:** supervision, writing – original draft, writing – review and editing.

## Disclosure

Guarantor: Susmin Karki is the guarantor of this case report.

## Ethics Statement

Our institution does not require ethical approval to report individual cases.

## Consent

Written informed consent was obtained from the patient to publish this report in accordance with the journal's patient consent policy.

## Conflicts of Interest

The authors declare no conflicts of interest.

## Data Availability

The data that support the findings of this study are available from the corresponding author upon reasonable request.
