# Correction to “Neuromyelitis Optica Mimicking Multiple Sclerosis: A Case Report and a Comprehensive Review of the Literature”

**DOI:** 10.1002/ccr3.73622

**Published:** 2026-09-28

S. Thatal , S. Karki , A. Parajuli , et al., “Neuromyelitis Optica Mimicking Multiple Sclerosis: A Case Report and a Comprehensive Review of the Literature,” Clinical Case Reports 13, no. 10 (2025): e71277. 10.1002/ccr3.71277 PMC1252162541103591

The name of the sixth author was misspelled and has been corrected from Dibasha Adhikari to Dibasa Adhikari in the author list and author contributions section.

The online version of this article has been corrected accordingly.

We apologize for this error.

